# Challenging behaviors in children with autism spectrum disorders and quality of life in their parents: association between challenging behaviors and parental quality of life

**DOI:** 10.3389/fpsyg.2026.1569087

**Published:** 2026-03-16

**Authors:** Nur Hidayah Yahya, Wai Wai Yang, Nur Azah Isa, Wan Syanaz Wan Ghazali, Norazlin Kamal Nor

**Affiliations:** 1 Hospital Tunku Ampuan Besar Tuanku Aishah Rohani, Hospital Pakar Kanak-Kanak Universiti Kebangsaan Malaysia, Kuala Lumpur, Malaysia; 2 Department of Paediatrics, Universiti Kebangsaan Malaysia Faculty of Medicine, Kuala Lumpur, Malaysia

**Keywords:** ASD-BPC, autism spectrum disorder, challenging behaviors, parental quality of life, QoLA, stereotyped behaviors

## Abstract

**Background:**

Autism spectrum disorder (ASD) is a complex neurodevelopmental disability that typically appears early in life. ASD is frequently associated with challenging behaviors (CBs), which can be categorized into aggressive or disruptive, stereotypic and self-harming behaviors. The prevalence and patterns of challenging behaviors in ASD children have not been reported in the Malaysian population. Challenging behaviors may influence caregiver demands and parental quality of life. Understanding how prevalent challenging behaviors are, the types of behaviors exhibited, what factors contribute to them, and how they affect the quality of life of parents with ASD children are important aspects of ASD management.

**Objectives:**

This study was conducted to determine the prevalence and types of challenging behaviors in children with ASD, and factors that predict them. The quality of life in parents of children with ASD, and predictors for these, were also assessed. Finally, the association between challenging behaviors and quality of life in parents was determined.

**Methods:**

In this purposive consecutive study, a total of 166 parents of children with ASD aged between 2 to 18 years old were recruited from the Child Development Centre (CDC) in Universiti Kebangsaan Malaysia over a period of 11 months between 1st June 2021 and 31st May 2022. The Autism Spectrum Disorders – Behavior Problem for Children (ASD-BPC) questionnaire was translated to the Malay language and used to assess prevalence and subtypes of challenging behaviors in ASD children. The quality of life of parents was ascertained using the Quality of Life in Autism (QoLA) questionnaire and described in median and IQR. Univariate and multivariate regression was performed to assess predictors for challenging behaviors and parental quality of life. Mann-Whitney test was utilized to determine the association between challenging behaviors and parental quality of life.

**Results:**

The overall prevalence of challenging behaviors in ASD children was 89.8%, with the highest prevalence seen for stereotypic behaviors (75.9%). The predictors for challenging behaviors were child age and family income. The median quality of life score in parents of ASD children in this study was 103.0 (IQR:25). Challenging behaviors in ASD children were found to be associated with parental quality of life, with lower quality of life scores observed in ASD children who have “aggressive/disruptive” behaviors (*p* = 0.041) and “self-injurious” behaviors (*p* = 0.042).

**Conclusions:**

The prevalence of challenging behaviors amongst ASD children was high (89.8%), of which the most prevalent type was stereotypic behavior. Presence of aggressive or disruptive and self-injurious behaviors were linked to lower quality of life in parents, highlighting the need for tailored support and interventions to improve family wellbeing.

## Introduction

### Autism spectrum disorder (ASD)

The Centres for Disease Control and Prevention (CDC) defines ASD as a complex neurodevelopmental disability caused by differences in the brain that typically appears early in life. Symptoms of autism may be detected in early childhood, but autism may not be diagnosed until later in age as mentioned in Clinical Testing and Diagnosis for Autism Spectrum Disorder (2025), Autism (2025).

The diagnosis of ASD is made clinically based on medical history and clinical observations of child social interactions and behaviors using the Diagnostic and Statistical Manual of Mental Disorders, Fifth Edition (DSM- 5) criteria for Autism Spectrum Disorder [Centers for Disease Control and Prevention (CDC), 2025]. This consists of two main features, which are persistent impairments in reciprocal social communication and interaction (SCI), and restrictive, repetitive patterns of behavior, activities or interests, and sensory issues (RRBs) manifested currently or by history. For each of the two core features, severity is classified into three levels based on impact on the child's functioning from mild (“Requiring support”), moderate (“Requiring substantial support”), to severe (“Requiring very substantial support”).

The abilities and needs of children with ASD vary and can evolve over time. While some children with ASD can live independently, others may require varying levels of support. ASD is frequently associated with co-occurring conditions, for example epilepsy, attention deficit hyperactive disorder, intellectual disability and learning disability (Bougeard et al., 2024) as well as behaviors that may need more support, such as aggressive behavior and self-injurious behavior (Edelson, 2021). Children with ASD exhibit a wide range of intellectual functioning, from significant support to above-average levels of cognition (Khachadourian et al., 2023).

The prevalence of autism worldwide is approximately 1 in 100 children [(World Health Organization Quality of Life (WHOQOL) Group, 1995)] with male-to-female ratio of 3-4:1 (Loomes et al., 2017). ASD prevalence has been increasing worldwide in the past few decades by over 200% (Maenner et al., 2023). Caring for a family member with Autism Spectrum Disorder (ASD) can fundamentally alter family dynamics and have been reported to affects caregivers' mental health, frequently leading to chronic stress, anxiety, and interpersonal difficulties. (Sánchez Amate and Luque de la Rosa, 2024). Vasilopoulou and Nisbet (2016) reported that parents of children diagnosed with ASD experienced higher parenting stress and depressive symptoms. Kuhlthau et al. (2014) found that parents of children with autism spectrum disorder had clinically significant lower health-related quality of life scores compared to the normative U.S. population, with 40% of parents reported having clinical depression symptoms. John et al. (2025) reported that higher parental stress, along with increased levels of child internalizing, externalizing, repetitive behaviors, and communication difficulties, were all found to be linked to lower parental Quality of Life (QoL) scores. Conversely, better adaptive functioning in autistic children was associated with a higher QoL for their parents. The families of autistic children play a central role in supporting their child's development, which can include addressing behavioral or health-related challenges (O'Nions et al., 2018).

Children with ASD are often referred for intervention by their caregivers. Examples of these interventions include: early intervention programme, occupational therapy including for joint attention, management of fine motor difficulties, sensory integration, and adaptive function support, speech therapy for speech impairment and social skills training, as well as behavioral interventions and parental counseling and support (Althoff et al., 2019). Early intervention is associated with better outcomes (Heyvaert et al., 2014). In addition, at times, pharmaceutical intervention may be introduced as part of therapy, for example: Risperidone in children with aggressive and self-harming behaviors, methylphenidate for ASD children with comorbidity of Attention Deficit Hyperactivity Disorder, or melatonin for sleeping difficulties (Santosh and Singh, 2016).

### Challenging behaviors

The formal definition of challenging behaviors (CBs) is abnormal behaviors of such an intensity, frequency or duration that the physical safety of the person or others is likely to be placed in serious jeopardy, or behavior which is likely to seriously limit use of, or result in, the person being denied access to ordinary community facilities (Emerson and Bromley, 1995). In ASD, CBs can be categorized into three subtypes: (i) Aggressive/disruptive behaviors, (ii) Stereotypic behaviors, and (iii) Self-injurious behavior.

The first subtype of CBs is aggressive/disruptive behaviors and includes physical aggression toward others for e.g., hitting, biting, kicking, scratching, pulling hair and pinching (Alink et al., 2006); property aggression for e.g., throwing objects, hitting or kicking objects and urinating or defaecating on objects (Crocker et al., 2006; Matson et al., 2009); verbal aggression for e.g., yelling or cursing at others, threatening to harm others and bullying (Hemmings et al., 2006); and finally, sexual aggression for e.g., masturbating in public or inappropriately touching others (Crocker et al., 2006).

The second class of CBs, stereotypic behaviors, is defined as non-functional repetitive motor and/or vocal behaviors (Matson and Nebel-Schwalm, 2007). Stereotypies form one of the core features in ASD. Motor stereotypy is the type of stereotypy most associated with ASD, including involuntary and patterned hand-flapping, body rocking, repetitive finger/arm movements, repetitive whole body movements and spinning (Mahone et al., 2004). Vocal stereotypies also occur, for e.g. repetition of words, phrases, or sounds in a context where repetition is not necessary such as humming, as well as immediate or delayed echolalia (Lanovaz and Sladeczek, 2012). Play stereotypy describes non-functional play behaviors such as lining up toys or spinning wheels. While stereotypic behaviors are often studied in association with challenging behavior, current interventions only address it if it impedes on independence or engagement in learning opportunities (Wunderlich et al., 2023).

The third class of CBs, self-injurious behavior, is defined as physical aggression toward oneself that results in tissue damage (Iwata et al., 1994). They described self-injurious behaviors with examples such as self-hitting with an open or closed hand, self-biting, self-scratching, banging one's head against another part of one's body or another surface, poking oneself in the eye, ingesting inedible objects (i.e., pica), self-kicking, and hair pulling.

Challenging behaviors in autistic children are often reported in clinical and educational contexts. From previous studies, challenging behaviors typically associated with ASD include aggression toward others, self-injurious (or self-harming) behaviors, and severe tantrums (Hampton et al., 2021). Jang et al. (2011) reported that 94% of their study sample had challenging behavior, with stereotypical behaviors being most frequently reported. They also found that ASD severity was a predictor of challenging behaviors. Nicholls et al. (2019) found in their study that 53% of ASD children had at least one challenging behavior, 36.4% had self-injurious behavior, 30.2% aggressive/destructive behavior, and 25.9% stereotyped behavior. Meanwhile Hill et al. (2014) found that 1 in 4 children who had ASD scored in the clinical range for aggressive behaviors. Recent studies shows that challenging behaviors in autistic children can also contributed by other factors, not only the severity of autism symptoms. Studies have linked the presence of intellectual disability and lower adaptive skills with a greater likelihood of these behaviors (Esteves et al., 2021). Graziosi and Perry (2023) found that age and sex contribute to variations in challenging behavior among autistic children. Younger age and female sex have been associated with higher levels of certain behavior problems, while lower developmental level is also linked to increased behavioral difficulties. These findings emphasize the importance of looking at a variety of developmental and demographic factors when trying to understand why challenging behaviors appear in autism.

### Quality of life (QoL)

World Health Organization Quality of Life (WHOQOL) Group (1995) defines Quality of life (QoL) as an individual's perception of their position in life in the context of the culture and value systems in which they live and in relation to their goals, expectations, standards and concerns.

Parenting a child with autism spectrum disorder, particularly one that is associated with challenging behaviors, can be difficult at times. The challenge of raising such a child can strain the parents' mental wellbeing and their quality of life (Lope et al., 2013).

The quality of life of parents needs to be considered and measured as it may impact the quality of care provided to their children (Khanna et al., 2011; Rizk et al., 2011), and their children's functioning and outcomes (Frazier et al., 2020). It may help both clinicians and researchers plan the best intervention that would be beneficial for both ASD children and their parents.

Parental support is an important factor that takes place in the process of raising an autistic child. Parental support and caregiver quality-of-life (QoL) were recognized as important factors in the developmental progression of children with Autism Spectrum Disorder (ASD) (Pardo-Salamanca et al., 2025). When parents receive good social support, participate in adaptive coping, and maintain good self-efficacy and wellbeing, their risk of distress will be reduced, which can buffer negative effects on child behavior and promote more positive parenting practices (Higgins et al., 2022).

Families of children with ASD frequently experience higher levels of parenting stress, associated with the child's behavioral problems, sleep difficulties, and emotional difficulties. Studies have shown that the quality of life (QoL) of parents who had children with ASD is comparatively lower than that of parents of typically developing children (Lope et al., 2013; Vasilopoulou and Nisbet, 2016). Turnage and Conner (2022) found that the protective factors for parental QoL were parental education level and the severity level of ASD in the child. Vasilopoulou and Nisbet (2016) stated that variables associated with lower parental QoL were child behavioral difficulties, unemployment, and lack of social support. Other factors found to be associated with higher quality of life scores were employment, higher income and parents living together, while primary caregiver role and use of medications (child) were associated with lower scores (Calonge-Torres et al., 2017). A study in Malaysia by Asahar et al. (2021) reported that factors associated with higher parental quality of life in Malaysia were staying in a single-story house, attending two or more parent training sessions per year and receiving help to care for a child with ASD.

However, there are very few published studies in Malaysia on the quality of life of parents with autistic children, and even fewer with specific quality of life measures. In addition to that, there are also only a few studies on challenging behaviors in ASD children in Malaysia, even from the more urban Klang Valley region. A recent study in Sarawak found a high prevalence of problem behaviors among Malaysian ASD children and significant caregiver burden (Chua et al., 2023). While there is an increase of ASD research globally, studies examining challenging behaviors and family impact among ASD children, especially in Malaysia, remain limited. The aim of this study was to address this gap, specifically: (1) to determine the prevalence and patterns of challenging behaviors among Malaysian ASD children in Klang Valley, which differs demographically and culturally from previous studies such as in Sarawak, (2) to explore how family support, parental stress, and local contextual factors (e.g., access to services or community support) relate to child behavior and family quality of life — factors which may influence both care-seeking and intervention outcomes.

Understanding CBs in ASD can improve our knowledge of autism presentation and improve strategies for intervention, thus helping to manage the child holistically and more effectively.

## Materials and methods

### Study design and population

This study was a cross-sectional study conducted in the Child Development Centre, Universiti Kebangsaan Malaysia (CDC UKM). Initially, participants were recruited at the CDC located within Hospital Canselor Tuanku Muhriz (HCTM, UKM), Bandar Tun Razak, Kuala Lumpur. On 1 March 2022, the pediatric services of UKM relocated to the newly established UKM Specialist Children's Hospital — Hospital Pakar Kanak-Kanak UKM, also known as Hospital Tunku Ampuan Besar Tuanku Aishah Rohani (HosTAR), which now houses the Child Development Centre. It was conducted within 11 months, from 1st June 2021 to 31st May 2022, covering both pre-move and post-move periods. Ethical approval and study protocols remained constant throughout. As the relocation did not interrupt clinical services nor alter diagnostic procedures, parents of autistic children and adolescents aged between 2 to 18 years who fulfilled the inclusion criteria were selected during this period.

Diagnosis of ASD for participant inclusion was based on prior clinical evaluation or consultation by a qualified developmental pediatrician at the Child Development Centre, UKM. Clinical diagnosis adhered to either the Diagnostic and Statistical Manual of Mental Disorders, Fourth Edition (DSM-IV) criteria or the Diagnostic and Statistical Manual of Mental Disorders, Fifth Edition (DSM-5), depending on when the assessment was made. For those diagnosed under DSM-5, severity levels for social communication impairment (SCI) and restricted, repetitive behaviors (RRBs) were determined via structured clinical assessment and consensus discussion by the developmental pediatrician, based on clinical history, parent interview, and direct behavioral observation.

The inclusion criteria were all parents of children and adolescents aged 2-18 years diagnosed with ASD by clinicians in CDC, parents who could communicate or read in the Malay or English languages and parents who consented to the study by written informed consent. The exclusion criteria were parents who could not communicate or read in the Malay or English languages. At recruitment, parents were asked whether they preferred to complete the questionnaire in English or in Malay (Bahasa Malaysia), and the corresponding paper-based version was provided to ensure that all participants could fully understand the items. Language preference was used solely for the purpose of administering the questionnaire and was not included as a variable in the statistical analyses. Parents of autistic children were selected using purposive consecutive sampling. Medical records of patients attending the CDC clinic within the study period were reviewed to identify those who met the inclusion criteria. Parents were recruited by the primary investigator with the help of staff nurses and doctors in the CDC clinic. Only one parent from each family were selected and given explanation regarding the purpose of the study. Regarding determination of the participating parent, there was no predetermined selection rule: if only one parent accompanied the child, that parent was invited to complete the questionnaire; if both parents were present, we asked which parent was primarily responsible for the child's daily care at home, and that parent was invited to participate. Once consented, a set of questionnaires in English or Bahasa Malaysia were given to parents (depending on which language parents were more comfortable with). The parent filled up the questionnaire while in the waiting room.

The questionnaire comprised of:

1) Socio-demographic data

The socio-demographic questionnaire consists of two sections. The parent section collects information on the child's gender, age, school attendance, birth order, number of siblings, parents' age and highest education, household income, parental health issues, and the child's medication use, including type and duration. Parental health information was obtained through a self-report item asking whether the mother or father had any existing medical comorbidities; no additional details regarding diagnosis, disease severity, or treatment follow-up were collected. The doctor section records ASD severity, including Social Communication Impairment (SCI) and Restricted, Repetitive Behaviors (RRB) levels, any co-occurring medical or developmental diagnoses (e.g., ADHD, intellectual disability, learning disability, etc), and whether the child has received interventions such as early intervention programs, occupational therapy, speech therapy, or consultation with a clinical child psychologist or autism counselor/educator.

2) Autism Spectrum Disorder- Behavior Problems for Children (ASD-BPC)

The ASD-BPC is and informant based assessment scale designed to assess behavioral problems in individuals with ASD. This questionnaire had 18 items divided into 3 categories: Aggressive/Disruptive behaviors (12 Questions), Stereotypic behaviors (3 Questions) and Self –Injurious behaviors (3 Questions). Respondents rate each item on a 3-point scale (0 = not a problem, 1 = mild problem, 2 = severe problem). The items composing the internalizing scale assess for self-injurious behaviors, stereotypies, inappropriate sexual behavior and other odd behaviors, while items in the externalizing scale assess for physical aggression, verbal aggression, property destruction, and tantrum-like behaviors. To obtain scores on the externalizing and internalizing scale, numbers for certain items specific to each were totalled. Subscale scores are calculated by summing the items within each category, and a total score is obtained by summing all items, yielding a range from 0 to 36, with higher scores indicating greater behavioral difficulties.

For the ASD-BPC questionnaire, attempts were made to obtain formal permission from the original authors to use the English version and to translate it into Malay; however, these attempts were unsuccessful. The translation was therefore carried out following standard procedures: the English version was translated into Malay and back-translated by two bilingual individuals. Face and content validity were reviewed by two content experts, and a pilot study with 20 parents using both the English and Malay version of ASD- BPC was conducted to assess clarity and comprehension. Internal reliability of the translated version was evaluated based on the pilot study results. Cronbach's alpha comparing English version and Malay translation: 90% (excellent internal consistency).

(iii) Quality of Life in Autism Questionnaire (QoLA)

The QoLA is a self-report scale that measures the quality of life of parents with ASD children. The scale was developed for parents of children aged 2–18 years old. The QoLA consisted of two subscales: (i) Part A to measure parents' overall perception of their quality of life and (ii) Part B to evaluate the degree to which children with ASD's specific difficulties affected their parents. Part A included 28 items where parents rate their level of perception of how much they experience the said statement (item) on a five-point Likert scale ranging from 1 (not very much) to 5 (very much). Four items are reversed scored (Items 2, 4, 17, 22) and were adjusted before computing the total scores. The total scores for Part A were 28–140, with a higher score indicating higher perceived quality of life. Meanwhile, Part B consisted of 20 difficulties that children with ASD typically present with and parents again rate how problematic they perceive the difficulties to be. The total scores for Part B range from 20 to 100, with a higher score demonstrating a lower impact of children's difficulties on parents. The scores for each subscale should be addressed separately since both scales reflect two different contexts related to parents of children with ASD's quality of life.

For all questionnaires and their translated Malay version, there were no issues with data ownership as the questionnaire were freely available and could be download easily from internet. The Autism Spectrum Disorder – Behavior Problems for Children (ASD-BPC) questionnaire was sourced from Matson et al. (2008), “*Reliability of the Autism Spectrum Disorder-Behavior Problems for Children (ASD-BPC)”*. The Quality of Life in Autism (QoLA) questionnaire was obtained from the study “*Conceptualization and Development of a Quality of Life Measure for Parents of Children with Autism Spectrum Disorder”* by Eapen et al. (2014) and permission was obtained from the authors to use the Malay version, adapted from Shamsudin et al. (2018) “*Translation, Cross-Cultural Adaptation and Validation of the Quality of Life in Autism Questionnaire (QoLA) from English into the Malay Language”*.

The sample size was determined based on a previous published study on challenging behavior in children with autism (Murphy et al., 2009). Based on these results, a n estimated sample size of 130 was calculated using the formula:


$$\begin{array}{l} n=\;[Np(1-p)]/[(d2/Z2*(N-1)+p*(1-p)]. \end{array}$$

N: population size. The estimated number of ASD patients under CDC UKMMC follow up is 300 children

Z: statistic for a level of confidence. Z value is 1.96 for the level of confidence interval 95%

d: is precision 0.5%

Margin of error: 20%

A total of 169 parents fulfilled the inclusion criteria and were recruited, however, there were 3 parents who did not complete the questionnaires, and those responses were discarded. Hence, the total study population collected were 166 parents who responded to the questionnaires within the study period and had complete data.

### Statistical analysis

Statistical analysis was performed using IBM SPSS statistics version 27. Cronbach's alpha was used to determine the internal reliability of the translated Malay version of the ASD-BPC. Demographic data was presented in median values with interquartile range or as frequency.

The significance level was set at *p* < 0.05. Descriptive analysis of CBs in ASD children and QoLA in parents of autistic children in CDC UKMMC was performed. Prevalences as well as individual items on each questionnaire were described as percentages. Predictors associated with overall and subtypes of challenging behaviors were analyzed using Fisher exact test for categorical data (except for income using univariate logistic regression) and Mann-Whitney statistical test for continuous data. The predictors that showed statistically significant association with challenging behaviors were then further analyzed using multivariate logistic regression.

Predictors associated with QoLA score total were analyzed using the Mann-Whitney statistical test for categorical data (except for income using Kruskal-Wallis statistical test) and simple linear regression for continuous data. The predictors that showed statistically significant association with quality of life were then further analyzed using multiple linear regression.

Multiple linear regression method was used to assess the association between parental quality of life and child's challenging behavior overall, and as stratified by type of challenging behavior.

### Ethical approval

Ethical approval was obtained from the Medical Research and Ethics Committee, Faculty of Medicine, Universiti Kebangsaan Malaysia to conduct the study at Child Development Centre (CDC) Universiti Kebangsaan Malaysia. Ethics Approval Code: FF-2021-255.

### Study demography: general

There was a total of 166 subjects in the study population.

The translated Malay version of the ASD-BPC was assessed for internal reliability using Cronbach's alpha. The Cronbach's alpha value was 90%.

### Study demography: child characteristics

Children with ASD ranged in age from 2 to 18 years, with a median age of 7 years (IQR: 5) years with the majority being boys (81.3%). Of these, 141 (84.9%) attended school. As shown in Table 1A, almost half of the participating patients (41.6%) had another comorbidity. Among them, ADHD and GDD/Intellectual disability were the most frequent, affecting 15.7% and 12.0% of the whole cohort respectively. Only 12 (7.2%) of the patients were on medication, and 7 (4.2%) were on medication for behavior, such as Methylphenidate or Risperidone.

**Table 1A:** Study demographic: child characteristics.

**Variables (*N* = 166)**	**Frequency, *n* (%)**	**Median (IQR)**
**Gender**		
Boy	135 (81.3)	-
Girl	31 (18.7)	
**Age (Years)**		7 (5)
**Attending school**		
No	25 (15.1)	-
Yes	141 (84.9)	
**Co-morbidities**		
No	97 (58.4)	-
Yes	69 (41.6)	
ADHD	26 (15.7)	
GDD/Intellectual disability	20 (12.0)	
Learning disability	8 (4.8)	
Others	22 (13.3)	
**Medication**		
No	154 (92.8)	-
Yes	12 (7.2)	
Medication for behavior^*^	7 (4.2)	
Other medication	5 (3.0)	
**Undergoing intervention**		
No	56 (33.7)	-
Yes	110 (66.3)	
Early intervention programme	49 (29.5)	
Occupational therapy	87 (52.4)	
Speech therapy	59 (35.5)	
Clinical child psychologist	12 (7.2)	
Autism counselor/educator	12 (7.2)	
**ASD severity (DSM 5)**		
**SCI**		
Level 1 (Mild)	78 (47.0)	-
Level 2 (Moderate)	68 (41.0)	
Level 3 (Severe)	11 (6.6)	
**RRBs**		
Level 1 (Mild)	104 (62.7)	
Level 2 (Moderate)	48 (28.9)	
Level 3 (Severe)	5 (3.0)	

IQR, Interquartile range; DSM, Diagnostic and Statistical Manual of Mental Disorders; SCI, Social Communication/Interaction Deficits; RRB, Restrictive and Repetitive Behavior; interests or activities.

^*^Medication for behavior (Methylphenidate, Atomoxetine, Risperidone), Level 1: Level 1 = mild (“Requiring support”), Level 2 = moderate (“Requiring substantial support”), and Level 3 = severe (“Requiring very substantial support”).

About two-thirds of the participating patients (66.3%) were undergoing intervention for ASD, and more than half (52.4%) had undergone regular occupational therapy. There were 9 older patients who were categorized using DSM IV, as they were diagnosed prior to publication of the DSM-5 version. SCI level 1 (mild) was the most common category of SCI severity with a frequency of 78 out of 166 (47.0%). For RRBs, most patients were categorized in RRB level 1 (62.7%).

### Study demographic: parent/family characteristics

Most of the questionnaires were completed by mothers (69.3%). The age of mothers ranged from 25 to 58 years (media*n* = 37 years, IQR = 7), and the age of fathers ranged from 28 to 64 years (media*n* = 37 years, IQR = 6). More than two-thirds of the parents had a tertiary education level (e.g., Diploma, Degree, etc.).

Financial-wise, the majority were categorized as M40 (middle family income) with 46.4%, while T20 (high family income) was the least with 17.5%. Most of the parents (95.2%) were married. Most respondents (71.7%) had 2-3 children in the house, while 24 (14.5%) had only one child. More than three-quarters of the parents had no previous medical diagnosis. Table 1B showed the sociodemographic and clinical characteristics of the parents.

**Table 1B:** Study demographic: parent/family characteristics.

**Variables (*N* = 166)**	**Frequency, *n* (%)**	**Median (IQR)**
**Form filler**		
Mother	115 (69.3)	-
Father	51 (30.7)	
**Parents' age**		
Mother age	-	37 (7)
Father age		37 (6)
**Parental highest education level attained**		
**Mother**		
Primary	2 (1.2)	
Secondary	46 (27.7)	
Tertiary	118 (71.1)	
**Father**		
Primary	2 (1.2)	
Secondary	51 (30.7)	
Tertiary	113 (68.1)	
**Family income**		
T20 (high)	29 (17.5)	-
M40 (middle)	77(46.4)	
B40 (low)	60 (36.1)	
**Marital status**		
Married	158 (95.2)	-
Divorced	8 (4.8)	
**Number of children in the nucleus family**		
1	24 (14.5)	-
2-3	119 (71.7)	
≥4	23 (13.9)	
**Medical diagnosis in parents**		
**Mother: No**	145 (87.3)	-
**Mother: Yes**	21 (12.7)	
DM	6 (3.6)	
Hypertension	5 (3.0)	
Others	16 (9.6)	
**Father: No**	139 (83.7)	
**Father: Yes**	27 (16.3)	
DM	9 (5.4)	
Hypertension	14 (8.4)	
Others	12 (7.2)	

IQR, Interquartile range; B40, M40 and T20: Family income of less than RM4850 (low family income), RM4850-10959 (middle family income) and more than RM10960 (high family income), respectively. DM, Diabetes Mellitus; Others: Hyperlipidaemia, Bronchial Asthma, Heart disease, Thalassemia, Cerebrovascular accident.

## Results

### Prevalence of challenging behaviors using the ASD- BPC questionnaire

Table 2 showed responses from parents according to type of challenging behaviors. Out of 166 respondents, 89.8% of children showed at least one challenging behavior using ASD-BPC. For each type of challenging behavior, 119 (71.7%) responded to at least one item in aggressive/disruptive behavior as a challenging behavior, 126 (75.9%) responded to at least one item in stereotypic behavior as a challenging behavior and 28 (16.9%) responded to at least one item in self-injurious behavior as a challenging behavior. Meanwhile, the most prevalent challenging behaviors was stereotypic behavior (75.9%) and the least prevalent was self-injurious behavior with only 16.9% respondents rating at least one item in this type as challenging behavior.

**Table 2:** Prevalence of challenging behaviors overall and by subtypes.

**Type of challenging behaviors**	**Ye *n* (%)**	**No *n* (%)**
Any challenging behavior	149 (89.8)	17 (10.2)
1. Aggressive/disruptive behavior	119 (71.7)	47 (28.3)
2. Stereotypic behaviors	126 (75.9)	40 (24.1)
3. Self-injurious behavior	28 (16.9)	138 (83.1)

Table 3 shows responses from parents for individual items in each type of challenging behavior. For aggressive/disruptive behaviors: banging on an object (e.g. door, wall) with a hand (34.3%) and property destruction (32.5%) were the most commonly reported items. The most challenging behavior rated as a severe problem for aggressive/disruptive behaviors was property destruction (6%).

**Table 3:** Frequency of challenging behaviors by individual items according to subtypes.

**Challenging behavior items**	**Responses from caregivers**			
	**Not a problem**, ***n*** **(%)**	**Mild problem**, ***n*** **(%)**	**Severe problem**, ***n*** **(%)**	**It is a problem**, ***n*** **(%)**
**1. Aggressive/disruptive behavior**				
Kicking object	123 (74.1)	41 (24.7)	2 (1.2)	43 (25.9)
Removal of clothes at an appropriate time	140 (84.3)	24 (14.5)	2 (1.2)	26 (15.7)
Inappropriate sexual behavior	146 (88.0)	16 (9.6)	4 (2.4)	20 (12.0)
Playing with their own saliva	131 (78.9)	33 (19.9)	2 (1.2)	35 (21.1)
Throwing objects at others	130 (78.3)	33 (19.9)	3 (1.8)	36 (21.7)
Banging on an object (e.g., door, wall) with a hand	109 (65.7)	54 (32.5)	3 (1.8)	57 (34.3)
Smearing or playing with feces	157 (94.6)	9 (5.4)	0 (0.0)	9 (5.4)
Leaving the supervision of the caregiver without permission (i.e., elopement)	120 (72.3)	43 (25.9)	3 (1.8)	46 (27.7)
Aggression toward others	128 (77.1)	33 (19.9)	5 (3.0)	38 (22.9)
Pulling others hair	142 (85.5)	23 (13.9)	1 (0.6)	24 (14.5)
Yelling or shouting at others	119 (71.7)	43 (25.9)	4 (2.4)	47 (27.3)
Property destruction (e.g., ripping, breaking)	112 (67.5)	44 (26.5)	10 (6.0)	54 (32.5)
**2. Stereotypic behaviors**				
Unusual play with an object (twirling strings, staring at toys)	82 (49.4)	74 (44.6)	10 (6.0)	84 (50.6)
Repeated and unusual vocalization (humming)	66 (39.8)	84 (50.6)	16 (9.6)	100 (60.2)
Repeated and unusual body movement (e.g., hand flapping)	73 (44.0)	78 (47.0)	15 (9.0)	93 (56.0)
**3. Self-injurious behavior**				
Poking him/herself in the eyes	161 (97.0)	4 (2.4)	1 (0.6)	5 (3.0)
Harming self by hitting/pinching/scratching	145 (87.3)	20 (12.0)	1 (0.6)	21 (12.6)
Mounting or swallowing objects causing body harm	151 (91.0)	15 (9.0)	0 (0.0)	15 (9.0)

Meanwhile, for stereotypic behavior, the most common items rated as challenging behavior were repeated and unusual vocalizations (60.2%) and repeated and unusual body movement (56%). When considering severity, repeated and unusual vocalizations (9.6%) was rated most highly as the most severe stereotypic behavior.

For self-injurious behaviors, the most reported items were harming self by hitting/pinching/scratching (12.6%) and mounting or swallowing objects, causing body harm (9%).

### Predictors for challenging behaviors

Univariate analysis was performed to determine association between specific predictors (child gender, child age, school attendance, parental educational level, family income, parental marital status, number of children in family, medical diagnoses in parents, co-occurring diagnoses in the child, co-occurring neurodevelopmental conditions in child) and challenging behaviors using Fisher exact test. The results are shown in Tables 4A, B. Age of the child was treated as a continuous variable in all analyses. The relationship between children age and each topography of challenging behavior (CB), including aggressive/disruptive, stereotypic, and self-injurious behavior was examined using univariate analysis. The factors that showed statistically significant association with aggressive/disruptive behaviors were female child gender, co-occurring diagnoses in the child and co-occurring neurodevelopmental conditions in child. Meanwhile, factors that showed statistically significant association with stereotypic behaviors were younger child age and family income (M40 vs. T20). Subsequently, factors that showed statistically significant association with self-injurious behaviors were younger child age and not attending school. Lastly, factors that showed statistically significant association with any challenging behavior was family income (M40 vs. T20).

**Table 4A:** Univariate analysis for predictor of challenging behavior.

**Variables**		**ASD-BPC (Part A: Aggressive/ disruptive behavior)**, ***N*** = **166**		***p* value**	**ASD-BPC (Part B: Stereotypic behavior)**, ***N*** = **166**		***p* value**	**ASD-BPC (Part C: Self -injurious behavior)**, ***N*** = **166**		***p* value**	**ASD-BPC (All items)**, ***N*** = **166**		***p* value**
		**Yes**, *n* **(%)**	**No**, *n* **(%)**		**Yes**, *n* **(%)**	**No**, *n* **(%)**		**Yes**, *n* **(%)**	**No**, *n* **(%)**		**Yes**, *n* **(%)**	**No**, *n* **(%)**	
Gender	Male	92 (68.1)	43 (31.9)	**0.045** ^**⋆**^	104 (77.0)	31 (23.0)	0.490	21 (15.6)	114 (84.4)	0.424	120 (88.9)	15 (11.1)	0.742
	Female	27 (87.1)	4 (12.9)		22 (71.0)	9 (29.0)		7 (22.6)	24 (77.4)		29 (93.5)	2 (6.5)	
Age, median (IQR)	7.0 (4.0)	8.0 (5.0)	0.226^b^	7.0 (4.0)	8.0 (5.0)	**0.014** ^**⋆*b***^	5.5 (5.0)	7.5 (5.0)	**0.021** ^**⋆*b***^	7.0 (4.0)	9.0 (6.0)	0.198^b^
School	Yes	21 (84.0)	4 (16.0)	0.157	104 (73.8)	37 (26.2)	0.202	19 (13.5)	122 (86.5)	**0.016** ^**⋆**^	125 (88.7)	16 (11.3)	0.474
	No	98 (69.5)	43 (30.5)		22 (88.0)	3 (12.0)		9 (36.0)	16 (64.0)		24 (96.0)	1 (4.0)	
Parent education	Secondary	21 (63.6)	12 (36.4)	0.283	23 (69.7)	10 (30.3)	0.368	4 (12.1)	29 (87.9)	0.604	29 (87.9)	4 (12.1)	0.749
	Tertiary	98 (73.7)	35 (26.3)		103 (77.4)	30 (22.6)		24 (18.0)	109 (82.0)		120 (90.2)	13 (9.8)	
Family income	B40 (low)	45 (75.0)	15 (25.0)	0.119^**a**^	42 (70.0)	18 (30.0)	0.647^**a**^	13 (21.7)	47 (78.3)	0.055^**a**^	53 (88.3)	7 (11.7)	0.137^**a**^
	M40 (middle)	57 (74.0)	20 (26.0)	0.127^**a**^	65 (84.4)	12 (15.6)	**0.037** ^**⋆*a***^	14 (18.2)	63 (81.8)	0.085^**a**^	74 (96.1)	3 (3.9)	**0.005** ^**⋆*a***^
	T20 (high)	17 (58.6)	12 (41.4)	Reference	19 (65.5)	10 (34.5)	Reference	1 (3.4)	28 (96.6)	Reference	22 (75.9)	7 (24.1)	Reference
Parent marital status	Married	114 (72.2)	44 (27.8)	0.689	120 (75.9)	38 (24.1)	1.000	26 (16.5)	132 (83.5)	0.623	142 (89.9)	16 (10.1)	0.587
	Divorced	5 (62.5)	3 (37.5)		6 (75.0)	2 (25.0)		2 (25.0)	6 (75.0)		7 (87.5)	1 (12.5)	

^⋆^Statistically significant at α = 0.05. Bold values indicate statistically significant results.

**Table 4B:** Univariate analysis for predictor of challenging behavior (Part 2).

**Variables**		**ASD-BPC (Part A: Aggressive/disruptive behavior)**, ***N*** = **166**		***p* value**	**ASD-BPC (Part B: Stereotypic behavior)**, ***N*** = **166**		***p* value**	**ASD-BPC (Part C: Self -injurious behavior)**, ***N*** = **166**		***p* value**	**ASD-BPC (All items)**, ***N*** = **166**		***p* value**
		**Yes**, ***n*** **(%)**	**No**, ***n*** **(%)**		**Yes**, ***n*** **(%)**	**No**, ***n*** **(%)**		**Yes**, ***n*** **(%)**	**No**, ***n*** **(%)**		**Yes**, ***n*** **(%)**	**No**, ***n*** **(%)**	
Total children in family	1	17 (70.8)	7 (29.2)	0.823	18 (75.0)	6 (25.0)	1.000	3 (12.5)	21 (87.5)	0.895	21 (87.5)	3 (12.5)	0.918
	2-3	84 (70.6)	35 (29.4)		90 (75.6)	29 (24.4)		21 (17.6)	98 (82.4)		107 (89.9)	12 (10.1)	
	≥4	18 (78.3)	5 (21.7)		18 (78.3)	5 (21.7)		4 (17.4)	19 (82.6)		21 (91.3)	2 (8.7)	
Parent medical diagnosis	Yes	31 (77.5)	9 (22.5)	0.423	29 (72.5)	11 (27.5)	0.672	10 (25.0)	30 (75.0)	0.145	37 (92.5)	3 (7.5)	0.765
	No	88 (69.8)	38 (30.2)		97 (77.0)	29 (23.0)		18 (14.3)	108 (85.7)		112 (88.9)	14 (11.1)	
Child co-occurring diagnosis	Yes	57 (82.6)	12 (17.4)	**0.009** ^**⋆**^	55 (79.7)	14 (20.3)	0.363	14 (20.3)	55 (79.7)	0.401	63 (88.7)	6 (8.7)	0.616
	No	62 (63.9)	35 (36.1)		71 (73.2)	26 (26.8)		14 (14.4)	83 (85.6)		86 (88.7)	11 (11.3)	
Neurodevelopmental conditions	Yes	42 (85.7)	7 (14.3)	**0.013** ^**⋆**^	38 (77.6)	11 (22.4)	0.844	9 (18.4)	40 (81.6)	0.821	45 (91.8)	4 (8.2)	0.780
	No	77 (65.8)	40 (34.2)		88 (75.2)	29 (24.8)		19 (16.2)	98 (83.8)		104 (88.9)	13 (11.1)	

^⋆^Statistically significant at α = 0.05. Bold values indicate statistically significant results.

Multivariate regression was performed with these factors. Using multivariate logistic regression stepwise method, two predictors were found to be associated with challenging behaviors: younger child age and middle family income (M40 vs. T20) as shown in Table 5. The child was less likely to have stereotypic behavior as the age increases. Younger child age was a predictor of stereotypic behaviors; for every year the child was older, the risk of stereotypic behavior reduced by 0.887 (OR 0.887, 95% C.I. 0.798-0.987, *p* = 0.028). We also found that family income was a significant predictor for stereotypic behaviors and for any challenging behaviors. A child from a family with income in the M40 (middle) category was 2.275 times more likely to have stereotypic behavior compared to a child from a family with income in the T20 (high) category (*p* = 0.044). A child from a family with income in the M40 (middle) category was also 7.747 times more likely to have any challenging behavior compared to a child from a family with income in the T20 (high) category (*p* = 0.006).

**Table 5:** Predictors associated with challenging behaviors: multivariate logistic regression.

**Factors**		**Aggressive/disruptive behavior**		**Stereotypic behavior**		**Self-injurious behavior**		**Any challenging behavior**	
		***p*** **value**	**Odd ratio (95% CI)**	***p*** **value**	**Odd ratio (95% CI)**	***p*** **value**	**Odd ratio (95% CI)**	***p*** **value**	**Odd ratio (95% CI)**
Gender	Male	Reference						Reference	
	Female	0.152	2.322	**-**	**-**	**-**	**-**	0.771	1.280
Age		**-**	**-**	**0.028** ^**⋆**^	0.887	0.301	0.922	0.412	0.933
School	Yes					Reference		Reference	
	No	**-**	**-**	**-**	**-**	0.081	2.656	0.469	2.354
Family income	B40			0.627	1.269			0.222	2.142
	M40	**-**	**-**	**0.044** ^**⋆**^	2.275	-	-	**0.006** ^**⋆**^	7.747
	T20			Reference				Reference	
Any co-occurring diagnoses	Yes	0.624	1.332					0.875	1.145
	No	Reference		**-**	**-**	**-**	**-**	Reference	
Co-occurring neuro developmental condition	Yes	0.320	1.978					0.583	1.713
	No	Reference		**-**	**-**	**-**	**-**	Reference	

^⋆^*p* value are statistically significant at α = 0.05. Bold values indicate statistically significant results.

### Quality of life in parents of children with autism

Tables 6, 7 shows parents' responses for each item in QoLA Part A and B. The quality of life scores were not normally distributed and thus non-parametric tests were used.

**Table 6:** Parental quality of life in parents of children with autism: (QoLA) Part A.

**QoLA Part A items**	**Scale (Very much of a problem for me) (Not much of a problem for me)**				
	**1**, ***n*** **(%)**	**2**, ***n*** **(%)**	**3**, ***n*** **(%)**	**4**, ***n*** **(%)**	**5**, ***n*** **(%)**
A1 Satisfied with life	3 (1.8)	6 (3.6)	51 (30.7)	76 (45.8)	30 (18.1)
**A2 Feel stressed**	5 (3.0)	17 (10.2)	50 (30.1)	66 (39.8)	28 (16.9)
A3 Feel happy and content	7 (4.2)	9 (5.4)	56 (33.7)	70 (42.2)	24 (14.5)
**A4 Feel depressed or anxious**	5 (3.0)	12 (7.2)	37 (22.3)	62 (37.3)	50 (30.1)
A5 Feel good about self or person	8 (4.8)	12 (7.2)	55 (33.1)	62 (37.3)	29 (17.5)
A6 Satisfied with a close relationship	4 (2.4)	5 (3.0)	41 (24.7)	74 (44.6)	42 (25.3)
A7 People are there for me when I need them	6 (3.6)	13 (7.8)	36 (21.7)	67 (40.4)	44 (26.5)
A8 Satisfied with social life	3 (1.8)	17 (10.2)	47 (28.3)	63 (38.0)	36 (21.7)
A9 Satisfied with family	5 (3.0)	6 (3.6)	28 (16.9)	66 (39.8)	61 (36.7)^#^
A10 Satisfied with the financial situation	7 (4.2)	18 (10.8)	66 (39.8)	53 (31.9)	22(13.3)
A11 Satisfied with where we live	5 (3.0)	8 (4.8)	36 (21.7)	70 (42.2)	47 (28.3)
A12 Enough money to meet needs	8 (4.8)	20 (12.0)	68 (41.0)	49 (29.5)	21 (12.7)
A13 Satisfied with achievements	5 (3.0)	16 (9.6)	66 (39.8)	63 (38.0)	16 (9.6)
A14 Satisfied with general health	7 (4.2)	13 (7.8)	50 (30.1)	59 (35.5)	37 (22.3)
A15 Have a healthy lifestyle	5 (3.0)	19 (11.4)	68 (41.0)	47 (28.3)	27 (16.3)
A16 Satisfied with leisure activities	8 (4.8)	21 (12.7)	58 (34.9)	55 (33.1)	24 (14.5)
**A17 Health problem stops them from doing things that they want to**	2 (1.2)	11 (6.6)	34 (20.5)	25 (15.1)	94 (56.6)^#^
A18 Feel in control of life	12 (7.2)	10 (6.0)	55 (33.1)	70 (42.2)	19 (11.4)
A19 Set and achieve goals in life	8 (4.8)	17 (10.2)	64 (38.6)	60 (36.1)	17 (10.2)
A20 Make a plan of action and follow it	7 (4.2)	18 (10.8)	47 (28.3)	78 (47.0)	16 (9.6)
A21 Make your own decision	4 (2.4)	14 (8.4)	43 (25.9)	81 (48.8)	24 (14.5)
**A22 Feel guilty**	3 (1.8)	16 (9.6)	40 (24.1)	61 (36.7)	46 (27.7)
A23 Part of a community	8 (4.8)	20 (12.0)	51 (30.7)	65 (39.2)	22 (13.3)
A24 Can get the support they need from the community	13 (7.8)^*^	17 (10.2)	56 (33.7)	62 (37.3)	18 (10.8)
A25 is able to get to where they need to	7 (4.2)	17 (10.2)	48 (28.9)	63 (38.0)	31 (18.7)
A26 Feel safe in everyday life	5 (3.0)	8 (4.8)	42 (25.3)	78 (47.0)	33 (19.9)
A27 Feel respected in everyday life	5 (3.0)	9 (5.4)	52 (31.3)	78 (47.0)	22 (13.3)
A28 Satisfied with the availability of health services	6 (3.6)	4 (2.4)	34 (20.5)	85 (51.2)	37 (22.3)

Bolded are reverse coded items.

^*^Items are the most frequently rated “very much of a problem.”

^#^Items are most frequently rated or “not much of a problem.”

**Table 7:** Parental quality of life in parents of children with autism (QoLA) Part B.

**QoLA Part B items**	**Scale (Very much of a problem for me) (Not much of a problem for me)**				
	**1**, ***n*** **(%)**	**2**, ***n*** **(%)**	**3**, ***n*** **(%)**	**4**, ***n*** **(%)**	**5**, ***n*** **(%)**
B1 Child socializing with people	22 (13.3)^*^	33 (19.9)	49 (29.5)	45 (27.1)	17 (10.2)
B2 Child having friends	17 (10.2)	38 (22.9)	46 (27.7)	38 (22.9)	27 (16.3)
B3 Child understand other's feelings	14 (8.4)	41 (24.7)	49 (29.5)	36 (21.7)	26 (15.7)
B4 Child holding a conversation	23 (13.9)^*^	42 (25.3)	44 (26.5)	40 (24.1)	17 (10.2)
B5 Child communicating needs	14 (8.4)	36 (21.7)	44 (26.5)	43 (25.9)	29 (17.5)
B6 Child taking a literal meaning of comments	21 (12.7)	37 (22.3)	45 (27.1)	34 (20.5)	29 (17.5)
B7 Child saying things that are socially embarrassing	7 (4.2)	8 (4.8)	29 (17.5)	31 (18.7)	91 (54.8)^#^
B8 Child needs to stick to a routine	5 (3.0)	13 (7.8)	47 (28.3)	36 (21.7)	65 (39.2)^#^
B9 Child being overly interested in a particular topic	7 (4.2)	13 (7.8)	48 (28.9)	41 (24.7)	57 (34.3)
B10 Child getting anxious in a specific situation or during changes	7 (4.2)	23 (13.9)	41 (24.7)	57 (34.3)	38 (22.9)
B11 Child is sensitive to certain sensations	7 (4.2)	19 (11.4)	47 (28.3)	48 (28.9)	44 (26.5)
B12 Child understands the rules of social interaction	17 (10.2)	34 (20.5)	46 (27.7)	44 (26.5)	25 (15.1)
B13 Child is able to manage emotional response	10 (6.0)	34 (20.5)	49 (29.5)	46 (27.7)	27 (16.3)
B14 Child needs to do things a certain way	6 (3.6)	26 (15.7)	49 (29.5)	48 (28.9)	37 (22.3)
B15 Child has destructive behavior, including anger and aggression	7 (4.2)	18 (10.8)	36 (21.7)	54 (32.5)	51 (30.7)
B16 Child showing inappropriate emotional reactions	4 (2.4)	18 (10.8)	38 (22.9)	54 (32.5)	52 (31.3)
B17 Child has unusual repetitive behaviors or body movement	4 (2.4)	19 (11.4)	34 (20.5)	55 (33.1)	54 (32.5)
B18 Child engaging in reckless or tactless behavior	5 (3.0)	16 (9.6)	27 (16.3)	55 (33.1)	63 (38.0)
B19 Child doing daily living tasks independently	14 (8.4)	27 (16.3)	34 (20.5)	39 (23.5)	52 (31.3)
B20 Child responding when approached socially	10 (6.0)	27 (16.3)	36 (21.7)	44 (26.5)	49 (29.5)

^*^Items are the most frequently rated “very much of a problem.”

^#^Items are most frequently rated or “not much of a problem.”

For QoLA Part A, which measures the parents' overall perception of their quality of life, the median score was 103.0 with IQR:25 (mean 101.63, SD ± 18.75), with a minimum score of 44 and a maximum score of 140. In QoLA Part A, the parents rated that “health problem stopping them from doing things they want” (56.6%) and “satisfaction with family” (36.7%) were the most frequently rated as “not much of a problem” for parents of children with ASD. On the other hand, “Can get the support they need from the community” (7.8%) was the item that was the most frequently rated as “very much of a problem” for the parents.

QoLA Part B measured parents' difficulties related to their child's ASD symptoms. The median score for QoLA part B was 70 (IQR: 25). The lowest score was 30, while the highest score was 100. For QoLA part B the items the parents rated as “very much of a problem” (or the worst problem) most frequently were “holding a conversation” (13.9%) and “socializing with people” (13.3%). Meanwhile, the items rated as “not much of a problem” (or the best) for parents regarding their child's ASD symptoms were the “child saying socially embarrassing things” (54.8%) and the “child needs to stick to a routine” (39.2%).

### Predictors for parental quality of life in parents of children with ASD (QoLA)

Univariate analysis was performed for predictors of parental quality of life QoLA Parts A and B. As shown in Table 8, by using the Mann-Whitney statistical test, Kruskal-Wallis statistical test and Bonferroni correction, there were three predictors that showed significant association with parental quality of life (QoLA Part A): higher parental education level (tertiary vs. secondary) (*p* = 0.006), parental married status (0.038) and high family income (B40 vs. T20) (*p* = 0.003).

**Table 8:** Univariate analysis: predictors associated with parental quality of life (QoLA) Parts A and B.

**Variables**		**QoLA (Part A)**		**QoLA (Part B)**	
		**Median (IQR)**	***p*** **value**	**Median (IQR)**	***p*** **value**
Gender	Male	107.0 (26.0)	0.079	71.0 (25.0)	0.291
	Female	94.0 (22.0)		68 (26.0)	
Age		*R*^2^ = 0.005	0.379^b^	*R*^2^ = 0.047	**0.005** ^**⋆*b***^
School	Yes	106.0 (27.0)	0.757	70.0 (25.0)	0.055
	No	100.0 (23.0)		57.0 (24.0)	
Parent education	Secondary	91.0 (22.0)	**0.006** ^**⋆**^	72.0 (19.0)	0.103
	Tertiary	106.0 (24.0)		68.0 (26.0)	
Family income	B40 (low)	96.0 (24.0)	**0.044** ^**⋆*a***^	69.0 (27.0)	0.837^a^
	M40 (middle)	106.0 (27.0)		71.0 (22.0)	
	T20 (high)	110.0 (23.0)		67.0 (35.0)	
Pairwise comparison	B40 - M40	-	0.436		
	B40 - T20	-	**0.003** ^**⋆*c***^		
	M40 - T20	-	0.094		
Parent marital status	Married	104.0 (26.0)	**0.038** ^**⋆**^	70.0 (26.0)	0.462
	Divorced	87.0 (42.0)		64.0 (14.0)	
Total children in family	1	103.0 (26.0)	0.781^a^	69.0 (25.0)	0.437^a^
	2–3	103.0 (26.0)		69.0 (26.0)	
	≥4	101.0 (26.0)		75.0 (28.0)	
Parent medical diagnosis	Yes	98.0 (24.0)	0.505	72.5 (27.0)	0.276
	No	106.0 (27.0)		68.5 (26.0)	
Child co-occurring diagnosis	Yes	100.0 (28.0)	0.592	67.0 (24.0)	0.060
	No	106.0 (24.0)		71.0 (26.0)	
Neurodevelopmental condition	Yes	100.0 (30.0)	0.785	67.0 (24.0)	0.088
	No	105.0 (24.0)		71.0 (26.0)	

^a^ from Kruskal-Wallis statistical test, ^b^ for simple linear regression and ^c^ for Bonferroni correction.

R^2^ is the coefficient of determination.

^⋆^Statistically significant at α = 0.05.

For QoLA part B, simple linear regression statistical test showed that child age was a predictor for parents' difficulties related to their child's ASD symptoms, with older child age associated with reduced parental difficulties' related to their child's ASD symptoms (*p* = 0.005).

For QoLA Part A, multivariate regression was performed with the three predictors from the univariate analysis: parental educational level, family income and marital status. Using multivariate linear regression stepwise method, two predictors were found to be associated with parental quality of life: tertiary parental education level (*p* = 0.018), and parental married status (*p* = 0.015) as shown in Table 9. The *r*^2^ result was 0.098, showing that 9.8% of change in parental quality of life was significantly contributed by higher parental education level and married status.

**Table 9:** Multivariate linear regression analysis: predictors for parental quality of life (QoLA Part A).

**Factors/variables**	***p*-value**	***t*-value**
Parent education	0.018^⋆^	2.391
Family income	0.297	1.047
Marriage status	0.015^⋆^	−2.464

^⋆^Statistically significant at α = 0.05.

*r*^2^ = 0.098.

### Association between challenging behaviors in children with ASD and parental quality of life

For QoLA part B, the presence of any challenging behavior (*p* < 0.001), as well as each individual subtype of challenging behaviors were associated with increased parents' difficulties related to their child's ASD symptoms. In addition, we assessed if ASD severity level was associated with parental quality of life and parents' difficulties related to their child's ASD symptoms (Table 10). We found that parents who have ASD children at mild level (“requiring support”) of severity perceived the impact of their children's difficulties as lower (*p* = 0.001).

**Table 10:** Association between ASD severity and parental quality of life scores.

**Variables**		**QoLA (Part A)**		**QoLA (Part B)**	
**ASD severity (DSM 5)**		**Median (IQR)**	***p*** **value**	**Median (IQR)**	***p*** **value**
SCI	Level 1 (mild)	108.0 (23.0)	0.076	75.0 (29.0)	< 0.001^⋆^
	Level 2 (moderate)	100.5 (27.0)		65.5 (24.0)	
	Level 3 (severe)	106.0 (28.0)		60.0 (14.0)	
RRB	Level 1 (mild)	107.0 (24.0)	0.076	73.0 (27.0)	< 0.001^⋆^
	Level 2 (moderate)	97.5 (30.0)		63.5 (19.0)	
	Level 3 (severe)	89.0 (22.0)		60.0 (19.0)	

^⋆^Statistically significant at α = 0.05.

Table 11 shows the association between the presence of challenging behaviors and parental quality of life scores. From our analysis, for parental quality of life (QoLA Part A), the presence of aggressive/disruptive behavior (*p* = 0.041) and self-injurious behavior (*p* = 0.042) were associated with significantly lower median scores for quality of life.

**Table 11:** Association between the presence of challenging behaviors and parental quality of life scores.

**ASD-BPC**		**QoLA: Part A, Median (IQR)**	**p value**	**QoLA: Part B, Median (IQR)**	**p value**
Presence of ASD-BPC	Yes	101.0 (26.0)	0.091	68.0 (23.0)	< 0.001^⋆^
	No	107.0 (34.0)		86.0 (25.0)	
Aggressive/disruptive behavior	Yes	100.0 (24.0)	0.041^⋆^	66.0 (23.0)	< 0.001^⋆^
	No	112.0 (23.0)		81.0 (23.0)	
Stereotypic behavior	Yes	101.0 (25.0)	0.609	67.0 (23.0)	< 0.001^⋆^
	No	104.0 (29.0)		81.0 (25.0)	
Self-injurious behavior	Yes	92.0 (30.0)	0.042^⋆^	60.0 (24.0)	0.002^⋆^
	No	106.0 (25.0)		71.0 (26.0)	

^⋆^Statistically significant at α = 0.05.

Finally, we also assessed if ASD severity was associated with challenging behaviors (Table 12). We found that those with ASD of moderate severity (“requiring substantial support”) for social communication impairments (SCI), (*p* = 0.030) and restrictive behaviors, interests and activities (RRBs) (*p* = 0.049) had more challenging behaviors compared to those with ASD of mild severity.

**Table 12:** Association between ASD severity and challenging behaviors.

**Variables ASD severity (DSM 5)**		**ASD-BPC (Part A: Aggressive/disruptive behavior)**, ***N*** = **166**		***p* value**	**ASD-BPC (Part B: Stereotypic behavior)**, ***N*** = **166**		**p value**	**ASD-BPC (Part C: Self -injurious behavior)**, ***N*** = **166**		***p* value**	**ASD-BPC (All items)**, ***N*** = **166**		***p* value**
		**Yes**, ***n*** **(%)**	**No**, ***n*** **(%)**		**Yes**, ***n*** **(%)**	**No**, ***n*** **(%)**		**Yes**, ***n*** **(%)**	**No**, ***n*** **(%)**		**Yes**, ***n*** **(%)**	**No**, ***n*** **(%)**	
SCI	Level 1 (mild)	48 (61.5)	30 (38.5)	**0.030** ^**⋆**^	54 (69.2)	24 (30.8)	0.090	8 (10.3)	70 (89.7)	0.098	65 (83.3)	13 (16.7)	0.110
	Level 2 (moderate)	56 (82.4)	12 (17.6)		58 (85.3)	10 (14.7)		16 (23.5)	52 (76.5)		64 (94.1)	4 (5.9)	
	Level 3 (severe)	9 (81.8)	2 (18.2)		8 (72.7)	3 (27.3)		3 (27.3)	8 (72.7)		11 (100.0)	0 (0.0)	
RRBs	Level 1 (mild)	68 (65.4)	36 (34.6)	0.056	74 (71.2)	30 (28.8)	0.118	17 (16.3)	87 (83.7)	0.974	88 (84.6)	16 (15.4)	**0.049** ^**⋆**^
	Level 2 (moderate)	41 (85.4)	7 (14.6)		41 (85.4)	7 (14.6)		9 (18.8)	39 (81.3)		47 (97.9)	1 (21.0)	
	Level 3 (severe)	4 (80.0)	1 (20.0)		5 (100.0)	0 (0.0)		1 (20.0)	4 (80.0)		5 (100.0)	0 (0.0)	

^⋆^Statistically significant at α = 0.05. Bold values indicate statistically significant results.

## Discussion

Challenging behaviors are frequently reported in children diagnosed with ASD and may be associated with parental quality of life. We assessed challenging behaviors in ASD children as there is little published local data regarding prevalence and patterns or types of challenging behaviors. Although studies of similar topics have been conducted, this study contributes to understanding regional patterns and factors, which may differ due to cultural, policy, or systemic differences. Malaysian parents may experience ASD-related challenges differently due to varying levels of stigma, and differences in access to early intervention and support services. In this study, the prevalence of challenging behaviors in children with ASD was found to be 89.8% (*n* = 166), with 149 out of 166 children showing at least one challenging behavior. A range of prevalence of challenging behaviors has been reported, which vary depending on tools used and sampling heterogeneity. Our findings are fairly consistent, though slightly lower than that reported by Jang et al. (2011), who found that 94% of ASD children had some form of challenging behavior using the same tool as in this study, the ASD–BPC. Murphy et al. (2009) used the Behavior Problems Inventory and found that 82% of ASD children (*n* = 144) displayed challenging behavior. Meanwhile, Nicholls et al. (2019) reported that 53% of participants (*n* = 321) met the criteria for challenging behavior by using the behavior problems inventory. Overall, this illustrates the reporting frequency of challenging behaviors in ASD children, and how variations in the tools used and sampled populations can affect reported rates. Such behaviors may influence the child's developmental progress but also the experiences of those around them. This may be due to a limit in children's participation in learning, which may increase parenting demands. CB such as aggression, self-injury, tantrums, and stereotypy may interfere with a child's participation in learning activities and therapeutic interventions, limiting opportunities for social, communication, and adaptive skill development. These behaviors also might create considerable challenges on parents, as higher CB severity has been consistently linked to increased parenting stress, poorer mental health, and lower quality of life (Chua et al., 2023).

In this study we investigated challenging behaviors based on behavior types. Challenging behaviors were categorized into three subtypes, which were aggressive/disruptive behaviors, stereotypic behaviors and self-injurious behaviors. In terms of specific types of challenging behaviors, we found that the most prevalent was stereotypic behavior, in which 75.9% of respondents report at least one item of challenging behavior of this type. This is similar to Jang et al. (2011) who found that repetitive and stereotypic behaviors were the most frequently and highly endorsed items amongst ASD children in their study. Matson et al. (2009) also reported repetitive and stereotypic behaviors as the most prevalent challenging behaviors in their cohort. Stereotyped behaviors include motor, verbal and play stereotypies. These behaviors constitute one of the core diagnostic behavioral features in ASD itself, and is part of the DSM-5 criteria for ASD. In fact, when considering individual items in the ASD-BPC, the two specific items that were most frequently reported by parents were repeated and unusual vocalization (humming) reported in 60.2% and repeated and unusual body movement (e.g. hand flapping) in 56.0%. Although these persistent behaviors are challenging for parents to manage, interestingly when assessed for association with quality of life, this subtype of challenging behavior was the only subtype that was not statistically significantly associated with lower parental quality of life, with median quality of life scores that was the highest compared to other challenging behavior subtypes. This may be due to parents becoming more familiar with these behaviors over time, and because such behaviors are often perceived as having fewer practical impacts compared to the other behaviors mentioned.

In relation to ASD severity, some studies reported that higher ASD severity may be associated to more frequent challenging behaviors. This study found that aggressive/disruptive behavior were more frequently observed in participants with greater social-communication difficulties. Interestingly, similar to Jang et al. (2011), in this study we also found that those exhibiting challenging behaviors overall were more frequently observed among participants with greater ASD severity. Non-verbal or minimally verbally children, and among autistic children who face difficulty with forming social relationships, challenging behaviors may emerge in the child due to frustration or as a form of communication.

When assessing for the least prevalent challenging behavior, self-injurious behavior as a subtype was found to have the lowest frequency in our ASD population and was only reported in 16.9% of our respondents. However, even though it wasn't very prevalent, it appeared to be impactful, and parents of those with self-injurious behaviors were found to ha significantly lower quality of life (*p* = 0.042). This shows how while some challenging behaviors are not as common, it can still have a substantial effect on the child as well as on his parents. Self-injurious behavior tend to be difficult for parents to accept and manage as it results in their child being injured. The distress from these behaviors likely contribute to lower quality of life in the parents of these ASD children. Bohadana et al. (2019) found that parents who had less injurious incidents of challenging behaviors in their ASD children tended to report greater quality of life.

For specific challenging behavior items overall, the most frequently reported behavior item was repeated and unusual vocalizations (stereotypic behavior) in 60.2% and the least frequently reported item was poking themselves in the eyes (self-injury behavior) in 3%. Matson et al. (2009) and Jang et al. (2011) similarly found repetitive and stereotyped behaviors were the most frequently reported challenging behavior. For the least frequently reported challenging behavior, our study finding was similar to Jang et al. (2011) which reported poking himself/herself in the eye as the least commonly endorsed item (2.4%).

We subsequently assessed factors that may predict challenging behaviors in ASD children. Following multivariate logistic regression, the factors that appeared to predict any challenging behaviors in our study was M40 (middle) family income (*p* = 0.006). For subtypes of challenging behavior, M40 (middle) family income (*p* = 0.044) and younger ages (*p* = 0.028) were associated specifically with stereotypic behaviors.

In relation to factors associated with challenging behaviors, M40 (middle income group) showed a higher association with certain challenging behaviors. Mayes et al. (2012) also found behavior problems were more commonly reported in the lower socioeconomic groups. Socioeconomic status can influence access to assessment and intervention services, which may shape how early behavior needs are identified and supported. Early assessment and intervention may help address early behavioral needs and guide families toward appropriate supports, though access may be easier for higher-income families compared with lower-income families relying on limited public programs. Surprisingly, we did not find a significant association comparing the B40 (low income group) to the T20 (high income group) for any challenging behaviors; this may be due to inadequate sample size as this study was mainly to assess prevalence of challenging behaviors, and was not adequately powered to ascertain predictors of challenging behaviors in subtypes of challenging behaviors. Another possibility is that the middle income group, which is the largest income group in our study, may include more of urban dual-income families. These families may be facing constraints with access to early intervention programs due to program cost or time limitations. The association between middle income status and stereotypic behaviors may similarly reflect reduced access to early and sustained behavioral interventions. Although income levels were examined, the study did not collect information on participants' area of residence. Consequently, we were unable to determine whether families were from urban or rural settings, which may influence access to services, caregiving demands, and interpretation of socioeconomic differences. This limitation should be considered when interpreting the findings.

Another factor that appeared to predict challenging behaviors in this study was the effect of child age. Younger age was associated with stereotypic behaviors (*p* = 0.028) and for every increase in age by year, the odds of stereotyped behaviors was reduced by 0.887. MacDonald et al. (2007) also reported more stereotypic behavior in younger children with ASD (age 2–4 years old) compared to typically developing children. From clinical observation, younger children tend to present with stereotypies such as handflapping, spinning, tiptoeing, and humming more frequently than older children. Increasing developmental maturity tends to reduce stereotyped behaviors, which generally decrease in frequency as a child ages and undergoes behavioral intervention. As children with ASD grow older, they may have had cumulative exposure with intervention, as well as more opportunities to learn and practice self-regulation skills. Many interventions and therapies aim to support emotional regulation, develop coping strategies and establish structured routines, which could help them manage challenging behaviors more effectively overtime (Montroy et al., 2016). All these are possible reasons why older children in this study display less stereotypic behaviors. Holden and Gitlesen (2006) reported that the prevalence of challenging behaviors increased with age, however in their study they assessed predictors of challenging behaviors as a whole rather than for subtypes, they used Challenging Behavior Survey, a different tool, and their study population included adults.

In this study, we also investigated quality of life in parents of children with ASD using the QoLA autism-specific questionnaire. The QoLA Part A construct measures the overall perceptions that parents have about their quality of life (Eapen et al., 2014). The overall mean score for Part A in this study was 101.63 ± 18.75. The level of quality of life reported by parents in this study was higher than in some recent studies. For example, Asahar et al. (2021) reported QoLA (Part A) mean score of 88.55 ± 17.25 (QoLA) and Due et al. (2017) found QoLA (Part A) mean score of 91.6 ± 13.7 (QoLA). High QoLA in this study suggests that parents in this study experience a better quality of life. Some possible reasons that we found were they were satisfied with their family (reported as not being a problem by over three-quarters of respondents) and the majority (70%) do not have health issues that affect their activities. Other than that, a possible explanation for the high QoLA score in this study was that more than 50% of the children in this study were categorized with mild ASD severity (level 1). Children under this category often present with fewer observable support needs in structured settings, which may influence how caregivers perceive and report their quality of life. We postulate that some cultural and religious practices in Malaysia may impact parental perception of their child's condition and their quality of life, for example accepting life's trials and tribulations as part of life's spiritual journey, and being patient with challenges and struggles in daily life. Beliefs and spirituality including prayer and other practices that support resilience, as well as community support for child-rearing and other types of aid may be associated with better parent resilience, this affecting how quality of life is reported. The impact on religion and spirituality in families of children with ASD likely vary, and further studies are needed to clarify this correlation. Membership of supportive groups such as religious groups as well as participation in cultural events, festivals and celebrations may help parents feel less isolated and more connected to their community, reducing stress and thus promoting wellness.

This study found two factors that predicted better quality of life (based on QoLA Part A), which were tertiary level of parent education and married status in the parents. This is consistent with Asahar et al. (2021) who reported higher parental education level was associated with better quality of life in parents of children with ASD. Higher parental education may be associated with greater access to information, resources, and early intervention services. This can influence parents' understanding of ASD and their approach to supporting their child (Summers et al., 2005). Autism is a complex neurodevelopmental condition. Parents' access to various resources may help them make informed decisions regarding their child's care. However, outcomes vary widely across families, and education alone does not determine the quality of support provided. With regards to parental married status, Hsiao (2018) found that parents of children with ASD who lived with a partner perceived higher levels of quality of life compared to parents who did not. Married parents may get support from their co-parenting partners and share parenting duties in taking care of their children; having a good support system generally improves one's mental health and boosts resilience in the face of adversity.

The QoLA Part B construct measures the main caregivers' perceptions of how problematic their child's autism-specific difficulties were for them (Eapen et al., 2014). The overall mean score of QoLA Part B in this study was 69.77 ± 16.19. The score of QoLA part B was similar to Eapen et al. (2014) at 68.52 ± 17.56, but higher than Asahar et al. (2021) at 56.55 ± 12.35. One factor found to be associated with higher QoLA (B) scores was older child age. A possible reason is that as a child gets older, he may attend school, have a regular schedule and have the benefit of intervention. This may influence the presentation of challenging behavious, though it may vary across families. As children with ASD grow older, they may develop better communication skills thus they may be better to express their needs and wants better. Some studies, for example Maemonah et al. (2021), reported a significant correlation between age and speaking ability in children with ASD. While we did not assess the child's communication abilities in this study, further research is warranted to explore how communication skills may relate to the presentation of challenging behaviors.

Finally, we assessed the association between challenging behaviors and parental quality of life. In this study we found that lower quality of life scores were associated with having ASD children that displayed “aggressive/disruptive” behaviors (*p* = 0.041) and “self-injurious” behaviors (*p* = 0.042). For parental perception of quality of life based on children's behaviors, all types of challenging behaviors were associated with low quality of life scores. Our findings are consistent with a study by Bekhet et al. (2012) who reported an association with parental wellbeing and behavioral challenges in children with ASD. While the direction of this relationship is complex and likely influenced by various factors, parental wellbeing and child behavior may be interrelated. Further research is needed to clarify how these factors influence each other over time.

### Limitations and future studies

This study was not powered for predictors of subtypes of challenging behaviors and predictors of parental quality of life. Although some predictors were found to be statistically significant for both parameters, the r2 value of 9.8% was low, which may be due to inadequate study population to assess this association robustly or that other factors that were not assessed in this study may contribute to parental quality of life.

In addition, as our study population involved children with a wide age-range, and the challenging behaviors involved may vary at different ages. Although age was analyzed as a continuous variable, the wide age range (2–18 years) remains a limitation, as developmental differences across this spectrum may influence the manifestation of challenging behaviors. While age was included in analyses for stereotypy, similar analyses for other CB topographies could be affected by age-related variability that may not be fully captured in a continuous analysis. These behaviors and their impact on parents' quality of life may differ. Another limitation is that this study used cross-sectional analysis, and thus some assessments were performed at different times in relation to ASD diagnosis. Other factors such as intervention type, intensity and duration of intervention may influence both challenging behaviors as well as parental quality of life. Other potentially important factors were not measured in this study, which may have influenced the findings. These include the intensity and duration of interventions received by the child, the time since ASD diagnosis, the child's verbal ability, the quality of social support available to the family, and cultural or religious practices that may affect parenting and perceptions of challenging behavior. While it was not feasible to capture all of these factors in the current study, future research should consider including these variables to provide a more comprehensive understanding of the factors influencing challenging behaviors and parental experiences in families of children with ASD.

For future studies, we recommend that the study be powered to assess for predictors as well as considering other factors, such as intervention duration and intensity. A longitudinal study assessing how challenging behaviors change with age in the same individual might also be helpful. The duration since diagnosis and the quality of social support are two factors that may be relevant that warrant future studies too.

### Conclusions and recommendations

In conclusion, in this study a high prevalence of challenging behaviors amongst ASD children of 89.8% was reported, of which the most prevalent type was stereotypic behaviors. Predictors of challenging behaviors were younger child age and middle family income. The quality of life in parents of ASD children in this study was higher or comparable to studies on parents with ASD children previously published, and tertiary parental education level and married status predicted higher quality of life. The presence of challenging behaviors, especially self-injurious and aggressive behaviors, were associated with lower quality of life in parents of ASD children. A few recommendations from this study is for the managing medical practitioner to be aware of and identify any challenging behaviors such as stereotyped and self-injurious behaviors in ASD children as this may impact the child's outcome. Parental quality of life is also affected by challenging behaviors and both the child and parents would benefit from early identification and good management of challenging behaviors. Future research should investigate the influence of cultural factors, urban vs. rural residence, and parents' employment status on challenging behaviors and parental quality of life in children with ASD. Additionally, interventions and support services for parents should be considered. This may include access to mental health support, respite care, and training in managing challenging behaviors, which can help reduce caregiver stress, improve wellbeing, and enhance the overall family environment for children with ASD.

## Data Availability

The original contributions presented in the study are included in the article, further inquiries can be directed to the corresponding author/s.
